# Prices and market power in mental health care: Evidence from a major policy change in the Netherlands

**DOI:** 10.1002/hec.4222

**Published:** 2021-01-27

**Authors:** Chiara Brouns, Rudy Douven, Ron Kemp

**Affiliations:** ^1^ Erasmus MC Rotterdam The Netherlands; ^2^ CPB Netherlands Bureau for Economic Policy Analysis Den Haag The Netherlands; ^3^ Erasmus University Rotterdam Rotterdam The Netherlands; ^4^ The Netherlands Authority for Consumers and Markets (ACM) Den Haag The Netherlands

**Keywords:** insurer market power, hospital market power, hospital insurer bargaining, mental health prices

## Abstract

In the Dutch health care system of managed competition, insurers and mental health providers negotiate on prices for mental health services. Contract prices are capped by a regulator who sets a maximum price for each mental health service. In 2013, the majority of the contract prices equaled these maximum prices. We study price setting after a major policy change in 2014. In 2014, mental health care providers had to negotiate prices with each individual health insurer separately, instead of with all insurers collectively as in 2013. Moreover, after a cost‐price revision, the regulator increased in 2014 maximum prices by about 10%. Insurers and mental health providers reacted to this policy change by setting most contract prices below the new maximum prices. We find that in 2014 mental health providers with more market power, that is, a higher willingness‐to‐pay measure, contracted significantly higher prices. Some insurers negotiated significantly lower prices than other insurers but these differences are unrelated to an insurers' market share.

## 1 INTRODUCTION

In response to growing health care costs, the health care system in the Netherlands was reformed in 2006 to a system of managed competition. Managed competition is based on competing health insurers who are intended to be prudent buyers of care on behalf of their enrollees. Health insurers compete on the basis of premiums and quality of care offered by their contracted providers. The prices for health care products are determined by a bargaining process between insurers and providers (Enthoven & van de Ven, 2007).

In the health care literature, a lot of research has been done on price competition in health care markets. Gaynor and Town (2012) and Gaynor, Ho, and Town (2015) provide excellent surveys. Most studies in the literature are about price variation across providers. A general finding in these studies is the positive correlation between hospital market power and price. For example, Cooper, Craig, Gaynor, and Van Reenen (2019) find that hospital prices in the United States are positively associated with indicators of hospital market power. Even after conditioning on many demand and cost factors, hospital prices in monopoly markets are 15.3% higher than those in markets with four or more hospitals. A major challenge in the literature is to show that these correlations are indeed causal. Many studies in health care market study price effects after hospital mergers. Generally, after a merger the market power of a hospital increases which allows the researchers to test whether this results in price increases after the merger. For example, Dafny, Ho, and Lee (2019) show that hospital mergers yield price increases of 7%–9% for inclusion in insurers' networks. Gowrisankaran, Nevo, and Town (2015) estimate a model of hospital‐insurer bargaining and predict higher prices after hospital mergers. Lewis and Pflum (2017) show that not only local market concentration but also being a system hospital may result in higher prices in the bargaining game. In the Netherlands, Halbersma, Mikkers, Motchenkova, and Seinen (2011) use aggregated hospital data and find also that the market shares of hospitals (insurers) have a significantly positive (negative) impact on the hospital price–cost margin. Roos, Croes, Shestalova, Varkevisser, and Schut (2019) compare price development of merger hospitals with a control group for individual hospital products. They find that price effects of a hospital merger in the Netherlands are positive or not significant, depending on the hospital locations, products, and health insurer.

Studies about empirical price variation across insurers are mainly related to the employer based insurance market in the United States. They find that insurers pay lower prices if they have a larger market share or are able to channel patients to lower priced providers. For example, Roberts, Chernew, and McWilliams (2017) find that insurers with market shares of 15% or more negotiated prices for office visits that were 21% lower than prices negotiated by insurers with shares of less than 5%. Melnick, Shen, and Wu (2011) find that concentrated insurers are better able to counteract the price‐increasing effects of concentrated hospital markets as long as health plan markets remain competitive. Sorensen (2003), finds that not market share but the ability of insurers to channel patients to lower priced hospitals is the main reason for lower hospital prices. Ho and Lee (2017) also find that reduced insurer competition in a market may also strengthen insurers' bargaining leverage when negotiating with hospitals, thus potentially reducing total payments to hospitals.

In this study, we study insurer‐provider contract prices for a very specific market, the Dutch mental health care market, which the Dutch government placed under a regime of managed competition in 2008. However, this process toward more competition went slowly and insurer‐provider bargaining about individual prices was essentially introduced for the majority of the providers only as from 2014. Mental health care is a special market as uncertainty and variation in treatments are greater than in other health care markets (Frank & McGuire, 2000), making it difficult for insurers to buy care from providers on the basis of volume or quality. Prices of mental health services may therefore be an (or the most) important determinant in contract negotiations. The lack of information and transparency in treating mental illnesses makes it questionable whether the market is suitable for managed competition. Therefore, the Dutch government did not, as for the majority of products in the hospital market, allow for free price negotiations between insurers and providers but capped the contract prices by setting maximum prices for each mental health service. Maximum prices set by the regulator may serve as a reference price in the negotiation process. If maximum prices are close to (or below) cost prices, or if all providers possess strong market power, then contract prices will be equal to the maximum price. In that case variation in market power cannot be measured as there is no price variation. However, if we observe contract prices well below the maximum prices as set by the regulator, then we may use price variation to study the balance of market power between insurers and providers.

This study adds to previous price competition studies in several ways. First of all, we employ unique Dutch data on negotiated contract prices between insurers and providers on mental health services. To measure causal price effects, we use as quasi experimental design not mergers but a major policy change.^2^ The policy change comprises two important changes. First, a change to more competition. Before 2014, only the largest regional insurer negotiated, on behalf of all other insurers, with mental health care providers. After 2014, all insurers were obliged to individually negotiate prices with all mental health care providers, allowing more competition between health insurers and providers. Thus, 2014 was in fact the first year that individual competition was implemented as each insurer‐provider pair had to set‐up contracts and to negotiate about prices. Second, an increase in maximum prices. Each insurer‐provider pair received also more room to negotiate as the regulator increased maximum prices with about 10%, after an extensive revision of cost prices (NZa, 2014).^3^ Since most insurers and mental health providers reacted to this policy change by setting most contract prices well below the new maximum prices, we believe that this policy change is an ideal setting to study the balance of market power. Essentially, all players were put in a new situation and had to set up new contracts with new contract prices. Moreover, regulated maximum prices were increased to such an extent that there was sufficient room to study whether price setting may be caused by market power. We perform our econometric estimation in a non‐standard way as the distribution of contract prices is capped by a maximum price. We use fractional regression models to account for the skewness in contract prices.

We use a large proprietary administrative dataset of claims data, including all contract prices, between Dutch health insurers and mental health care institutions for the years 2013 and 2014. From the data, we construct market power measures, that is market share indicators for insurers and “willingness to pay” (WTP) indicators for providers. The WTP measures the added value of the healthcare provider to the insurer's network of providers (Capps, Dranove, & Satterthwaite, 2003). A higher WTP reflects a stronger bargaining position for the provider. Dranove and Ody (2016) demonstrated the theoretical and empirical value of the WTP over other measures of market power like Elzinga‐Hogerty based market shares of the provider. We use the WTP‐measures to explain contract prices in both years.^4^

Our main result is that mental health providers with a higher WTP have significantly higher contract prices. The fact that the majority of contract prices in 2014 are set below the maximum price provides evidence that insurer market power plays a role, especially if providers have low market power. However, we do not find that insurers' market shares have a significant effect on contract prices, suggesting that more aspects play a role than only market share. Presumably, some large insurers are less price oriented than others.^5^ This finding may be partly related to the fact that all large Dutch insurers are not‐for profit, who may pursue other objectives besides price maximization (Dafny, 2019).

Thus, our results confirm the general findings in this literature on the relation between provider concentration and prices in the hospital market and we expand this finding to the mental health care sector.

This paper is organized as follows. In Section 2, we describe the Dutch mental health care sector. In Section 3, we describe our regression model and our market power indicators. In Section 4, we present the data and descriptive statistics. In Section 5, we present the regression results and robustness tests. Section 6 concludes.

## 2 THE DUTCH MENTAL HEALTH CARE SECTOR

With the introduction of the Health Insurance Act (HIA) in 2006, a system of managed competition was introduced in the Dutch health care system. The main objective of this system is to guarantee consumers' access to good quality health care at a good price. For managed competition to be effective, many preconditions have to be fulfilled, such as free choice of insurer, risk‐bearing buyers and sellers, adequate product classification and pricing system, consumer information, contestable markets, freedom to contract, and transparency (Heijink, Mosca, & Westert, 2013).

During our period of research, 2013 and 2014, there were nine health insurance companies active, which we will call insurers in the rest of the paper.^6^ Five insurers purchased care separately, under which the largest four who have a combined market share of about 90% of the total market. Four smaller insurers purchased care together through a purchasing combination.^7^ In this paper we, therefore, study contract prices of six different insurers.^8^

The Dutch mental health care system is divided into curative and long‐term care. Curative mental health care is the subject of this paper and consists of primary and secondary mental health care. Primary mental health care is for short treatments and secondary mental health care for longer and more complex treatments that last up to a year.

Curative mental health care is part of the HIA since 2008. Before 1 January 2008, all mental health care was publicly financed through the Exceptional Medical Expenses Act. The inclusion of mental health care in the HIA was supported by the introduction of a new product classification system (Diagnosis Treatment Combinations or DTCs^9^) that is used for reimbursing providers for the different mental health services. A DTC consists of all activities that are performed and the time (in minutes) carried out for these activities. DTCs in mental health care consist of different product groups that cover different amounts of time measured in ranges of minutes, for example, “depression 250 till 799 min” or “anxiety 12.000 till 17.999 min”. Each DTC has its own price. In general, a DTC that covers a higher minute range is more expensive than a DTC that covers less minutes. After the DTC is closed, the insurer pays the contracted price to the provider. These contract prices may differ across providers but are capped by the regulator who sets a maximum price for each DTC.^10^ This maximum price is meant to cover average estimated labor and capital costs for a mental health service.^11^

In this study, we study only mental health care providers who work in large regional institutions that account for about 90% of all curative mental health services (NZa, 2013a).^12^ These providers range in size and can be regional providers for ambulatory care, but also specialized psychiatric hospitals. In these providers often many different types of mental health care specialists work together.

Mental health providers negotiate with health insurers about DTC‐prices and an annual budget that serves as ceiling.^13^ Mental health care providers receive the negotiated DTC‐price for every DTC they produce until they hit the annual budget. In principle, providers do not receive additional payments if they produce over the budget (NZa, 2013a).^14^ In practice, however, contracts may sometimes be supplemented with additional budget for example if the number of patients turns out to be larger than ex‐ante expected.^15^ Mental health care providers/insurers have an incentive to negotiate higher/lower DTC‐prices, as less/more production is needed to hit the annual budget.

Until 2013, providers did operate under a “representation model.” Each provider negotiated only with one representative health insurer, that is, the dominant health insurer in the so‐called geographical GHOR‐region.^16^ This dominant health insurer negotiated on behalf of all other health insurers, and was required to contract all mental health care providers in a region.^17^ Thus, in 2013, the contract price for a DTC did not differ within a provider for the different insurers. However, DTC prices between providers in a region were allowed to differ, because providers are not allowed to negotiate collectively, but had to negotiate with the dominant insurer individually (NZA, 2013a).

Important for our analyses are major policy changes that took place in 2014. In 2014, the “representation model” was abolished and all mental health care providers were placed under a regime of managed competition. Health insurers were already used to this type of competition as this was introduced in 2006 for other parts of curative health care (see e.g., Schut & Varkevisser, 2017). An important aspect of managed competition is (the threat of) excluding providers from the insurer network. Although preferred provider arrangements are generally slow in developing, in mental health care insurers started experimenting with not contracting specific treatments from large mental health institutions as of 2015 (NZa, 2015). Moreover, managed competition increased the room for market powers to negotiate on prices, quality and budgets, as all providers had to negotiate with all insurers individually.^18^ Thus each provider‐insurer pair had to negotiate their own contract prices for DTCs and, therefore, the prices for the same DTC may not only differ across providers but also per insurer within the same provider. Another major policy change in 2014 was a recalculation of cost price of the maximum prices by the regulator which resulted in an average increase of maximum DTC prices of about 10% (NZa, 2014).^19^

Both major policy changes were to a large extent exogenous for providers and insurers and have likely increased the room for competition. First, the change to more competition in 2014 increased the possibilities and incentives for individual insurers to purchase care on behalf of their own enrollees. Moreover, the dominant insurer in a region had to act like a representative insurer with fewer competitive incentives, because lower negotiated prices would apply for all health insurers in the region.^20^ Moreover, the increase in the maximum price increased the room to negotiate lower prices than the maximum price. Indeed (not all) individual health insurers “automatically” updated their prices to the maximum price, and (not all) health care providers were able to negotiate the maximum price for their DTCs. The policy change therefore allows us to study whether market power has played a role in this change in price setting. Did insurers with more market power negotiate lower prices and did health care providers with more market power negotiate higher prices?

## 3 THE MODEL

In this section, we will first describe the main variables, such as insurer and provider market power, and lay out the model. For understanding the price responses by health insurers and providers, we will estimate a reduced form model for 2013 and 2014. Some recent examples of structural bargaining models are Ho (2009) and Gowrisankaran et al. (2015). Although our main interest is measuring price responses in 2014, for comparison reasons, we model 2013 as well.

Since contract prices are capped by maximum prices, we construct a price_index variable, which is the relative distance of the maximum price minus the contracted price:

$${\text{price\_index}}_{dijt}=\frac{{\text{maximumprice}}_{dt}-{\text{contractedprice}}_{dijt}}{{\text{maximumprice}}_{dt}}$$

where *d* refers to a DTC, *j* to the provider, *i* to the insurer and *t* to the year. A value of zero implies that the price equals the maximum price. A deviation from zero can be interpreted as that insurers and providers have negotiated a discount on the regulated maximum price. Insurers and providers negotiate the same price for all DTCs within a primary diagnoses and treatment duration range. Therefore, we will use such a group of DTCs as unit of observation in our estimations, and we will use the number of observations within each group as weight in our regressions.^21^

To measure market power of the provider, we use the “WTP” method developed by Capps et al. (2003). The WTP is derived from a theoretical bargaining situation that takes place between the insurer and the mental healthcare provider. The advantage of this approach to more ad hoc measures like market share or HHI is that the WTP is theoretically founded and it does not need a specific market definition (Dranove & Ody, 2016). It captures the value of mental healthcare providers to an insurers' provider network. Since the market changed profoundly after the policy reform and thereby the position of the provider and insurer, we construct two different WTP measures.

In 2013, the WTP is based on the total production of a provider:

$${\text{WTP}}_{j}^{2013}=\underset{m}{\sum }{\text{WTP}}_{mj}^{2013}=\underset{m}{\sum }w_{mj}\frac{\text{ln}\left(\frac{1}{1-s_{mj}}\right)}{s_{mj}}$$

In this formula, *m* indicates the different micro‐markets, *s*_*mj*_ the market share of provider *j* in micro‐market *m*. The relative importance *w*_*mj*_ reflects provider's *j* market share of total production of all providers in micro‐market *m*. We define a micro‐market as a 4‐digital postal code, of which there are about 4800 in the Netherlands. In general, mental health care provider *j* will have a higher willingness‐to‐pay if they dominate many micro‐markets.^22^

We take into account that preferences and the geographic distributions of insurers' enrollees can differ across insurers, resulting in different WTP's across insurers.^23^ Therefore, we construct a willingness‐to‐pay measure per provider‐insurer pair for 2014:

$${\text{WTP}}_{ij}^{2014}=\underset{m}{\sum }{\text{WTP}}_{mij}^{2014}=\underset{m}{\sum }w_{mij}\frac{\text{ln}\left(\frac{1}{1-s_{mij}}\right)}{s_{mij}}$$

where ${\text{WTP}}_{ij}^{2014}$ is similar to ${\text{WTP}}_{j}^{2013}$ but *s*_*mij*_ and *w*_*mij*_ are now the market share of provider *j* in micro‐market *m* related to insurer *i*.

Also for the insurers' market power we will construct two measures, one before and one after the policy change. Before the policy change, in 2013, the insurance market operated under the ‘representation model’. In this model the representative and largest insurer in a region negotiates, on behalf of all other insurers, with all providers that operate in that region. Thus, a price for a DTC of provider *j* in that region is the same for all insurers. Moreover, we assume that the market power of an insurer is the same for all providers. Our market power indicator for the representative insurer *i* in region *k* is given by:

$$M_{ik}^{2013}=\frac{N_{ik}}{N_{k}}$$

where *N*_*ik*_ is the total number of DTCs reimbursed by the representative insurer *i* in region *k*, and *N*_*k*_ the total number of DTCs by providers in region *k*. The idea behind this measure is that the larger the representative insurer is within the region, the bigger its interests and incentives are to negotiate firmly, as fewer profits are spilled over to competitors. As the representative insurer has to act on behalf of all other insurers and purchase sufficient volume to meet the total market demand in the region, we include also the observations of the other insurers in the regression to obtain estimations that cover total market demand. As these insurers are smaller, their weighted effect in the estimation is also smaller.^24^

After the policy change, in 2014, the “representation model” was abolished for an individual competition model. In the competition model, we assume that each insurer‐provider pair in a region designs contracts and prices for DTCs with each provider in the market. Thus, variation in prices increases as DTC prices are now set for each insurer‐provider combination in the market. We measure market power for each individual insurer *i* as its market share within provider *j*:

$$M_{ij}^{2014}=\frac{N_{ij}}{N_{j}}$$

where *N*_*ij*_ is the total number of DTCs reimbursed by insurer *i* to provider *j* and *N*_*j*_ the total number of DTCs of provider *j*. The idea behind the measure is that an insurers' interest and incentive to negotiate firmly becomes stronger the larger its market share is within this provider.

Contract prices may also be influenced by underlying costs of a provider. We will use several variables that are related to costs as control variables in our model. A first one is the number of diagnosis categories, CAT_*j*,*t*_, provided by provider *j* in year *t* = 2013, 2014.^25^ A second cost variable is the share of complex patients, COM_*j*,*t*_, by provider *j* in year *t*, where we define a complex patient as a patient that is also treated by another provider than provider *j* during a year. A third measure is the share of patients of provider *j* that have treatments in more than one diagnosis category during a year MOR_*j*,*t*_, which can be interpreted as a measure for comorbidity. We use these control variables as a cost indicator for a provider. A provider with more problematic patients, that is, higher CAT_*j*,*t*_, COM_*j*,*t*_, MOR_*j*,*t*_ will generally have higher fixed and variable costs. We assume that mental health care providers use the same internal cost prices across insurers and regions in their price calculations.^26^

The dependent variable price_index is restricted by zero and one, where outcomes at the endpoints are allowed (0≤price_index≤1). We use a fractional regression model, introduced by Papke and Wooldridge (1996), which is used in numerous studies (see Ramalho, Ramalho, & Murteira, 2011 for a review).^27^ We assume a non‐linear conditional mean model for *price*_*index* as follows:

$$E\left({\text{price\_index}}_{dijt}|X_{dijt}\right)=G\left(X_{dijt}\beta \right)$$

where *G* is the cumulative logistic distribution function that has values between zero and one and may also be equal to 0 or 1. β contains the parameters of interest and can be consistently estimated with non‐linear least squares.^28^ We specify $X_{dijt}\beta$ separately for *t* = 2013 and *t* = 2014 as follows:

$$X_{dik}^{2013}\beta =\beta_{1}{\text{WTP}}_{j}^{2013}+\beta_{3}M_{ik}^{2013}+C_{j,2013}+D_{i}+D_{d}+\varepsilon_{dik}$$

$$X_{dij}^{2014}\beta =\beta_{2}{\text{WTP}}_{ij}^{2014}+\beta_{4}M_{ij}^{2014}+C_{j,2014}+D_{i}+D_{d}+\varepsilon_{dij}$$

where ${\text{WTP}}_{j}^{2013}$, ${\text{WTP}}_{ij}^{2014}$, $M_{ik}^{2013}$ and $M_{ij}^{2014}\;$represent the provider and insurer market power measures in 2013 and 2014. If provider market power plays a role in both years then we expect that$\;\beta_{1},\beta_{2}<0$, that is, the larger provider market power the closer the contract price will be to the maximum price and the closer the price_index variable will be to zero. In a similar way, if insurer market power plays a role in both years, we expect that$\;\beta_{3},\beta_{4}>0$. The larger the market power of an insurer, the larger the price index, that is, the price will be substantially lower than the maximum price. For the controls holds$\;C_{j,t}=\;\beta_{5}{\text{CAT}}_{j,t}$ $+\;\beta_{6}{\text{COM}}_{j,t}$ $+\;\beta_{7}{\text{MOR}}_{j,t}.$ As we have explained above we expect $\beta_{5},\beta_{6},\beta_{7}<0$, when providers cost prices increase contract prices will be set closer to the maximum price. Finally, *D*_*i*_ and *D*_*d*_ are insurer and diagnosis‐fixed effects. These fixed effects are used as control variables. *D*_*i*_ captures individual insurer characteristics, such as general administrative and quality aspects of insurers, but may also capture general insurer characteristics.^29^ *D_d_* captures general DTC aspects, such as the general cost price level. Finally, $\varepsilon_{dik}$ and $\varepsilon_{dij}$ represent the error terms that we cluster over providers and weight with the number of observations within each group. We run several robustness checks where we in‐ and exclude the insurer and diagnosis dummies, and the weights in the error term.

## 4 DATA AND DESCRIPTIVE STATISTICS

We use proprietary insurance claim data that includes all DTCs in the Netherlands between insurers and health care providers for 2013 and 2014.^30^ For each DTC, we have information about the contract price that an insurer pays to a mental health care provider. Each DTC contains information about the type of insurer, provider, and diagnosis category. Next, we computed groups of DTCs, where each group is uniquely determined by a combination of a diagnosis, a provider and an insurer.^31^ For both years we included all six insurers (five independent insurers and one purchasing combination) and all mental health providers, 164 in 2013 and 145 in 2014.^32^ We will use each group as unit of observation for our estimations and use the number of DTCs within each group as weight in our regressions.

Table 1 presents a summary of the main statistics. The sample size is respectively 21.603 observations for 2013 and 18.400 for 2014. This difference in size is mainly related to lower number of providers and some changes in the basic benefit package in 2014, when some diagnoses were transferred to primary mental health care (NZa, 2014).

**TABLE 1:** Summary statistics 2013 and 2014

	No. of obs.	Mean	st.dev	min	Max
**2013**					
price_index	21,603	0.016	0.035	0.000	0.973
${\text{WTP}}_{j}^{2013}$ (‘Willingness to pay’ measure)	21,603	1.167	0.168	1.002	1.582
$M_{ik}^{2013}$ (Market share largest insurer)	21,603	0.454	0.117	0.278	0.760
CAT_*j*, 2013_ (# diagnosis categories)	21,603	11.731	2.127	1.000	13.000
COM_*j*, 2013_ (complexity)	21,603	0.097	0.062	0.000	0.875
MOR_*j*, 2013_ (comorbidity)	21,603	0.093	0.041	0.000	0.400
**2014**					
price_index	18,400	0.060	0.055	0.000	0.991
${\text{WTP}}_{ij}^{2014}$ (‘Willingness to pay’ measure)	18,400	1.263	0.262	1.002	2.944
$M_{ij}^{2014}$ (Market share insurer in provider)	18,400	0.229	0.199	0.000	0.955
CAT_*j*, 2014_ (# diagnosis categories)	18,400	11.525	2.202	1.000	13.000
COM_*j*, 2014_ (complexity)	18,400	0.081	0.066	0.000	0.670
MOR_*j*, 2014_ (comorbidity)	18,400	0.080	0.041	0.000	0.400

**FIGURE 1:**
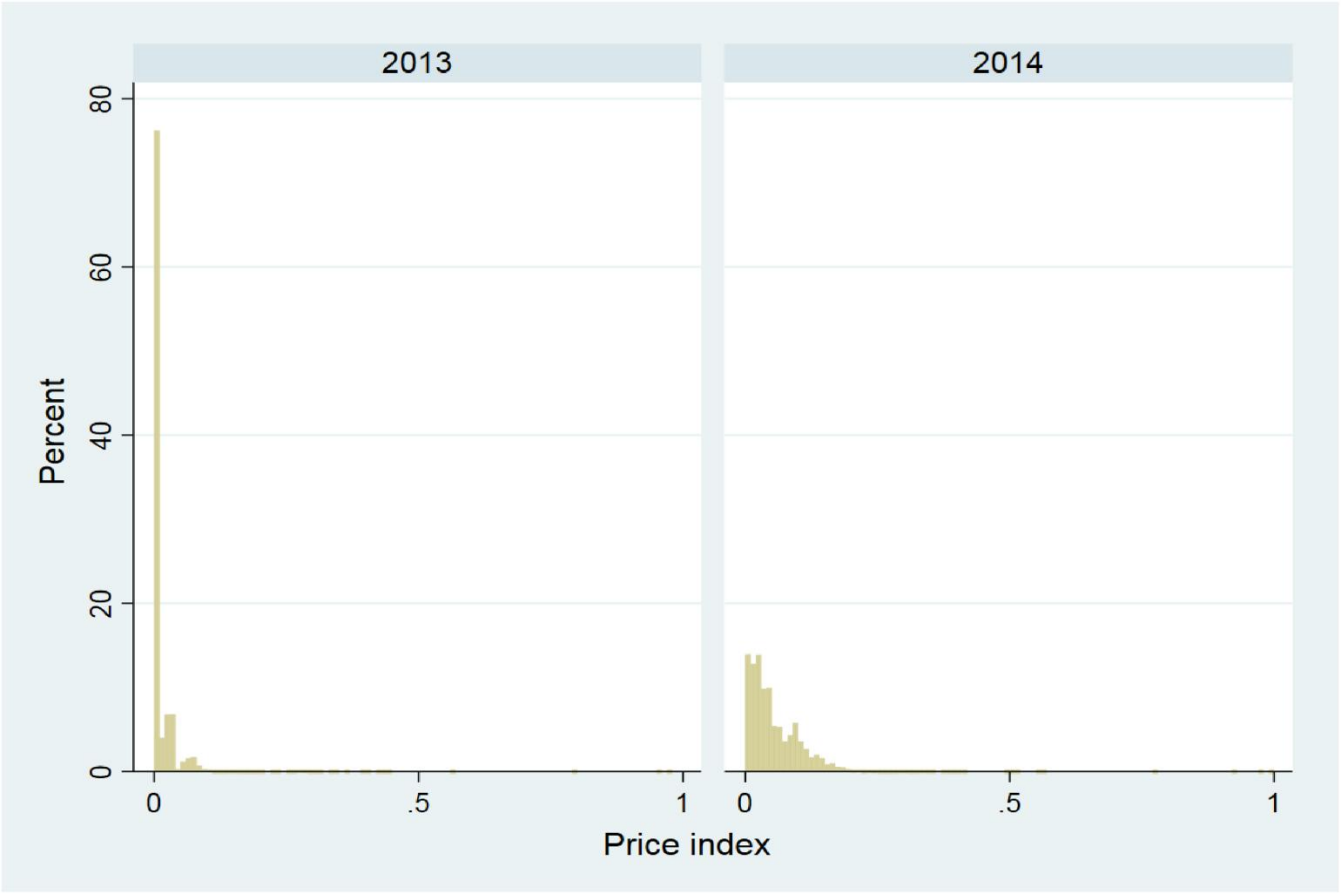
Distribution of price_index for the year 2013 and 2014. The figure shows the distribution of the price_index variable weighted with the number of observations in each group for 2013 (left panel) and 2014 (right panel). We have used a bin‐size of 0.01. As a result, the first bin in both panels contains also non‐zero values

The mean of the dependent variable “price_index” increases from 0.016 in 2013 to 0.060 in 2014, which suggests that the difference between the maximum price set by the regulator and the contract price has increased. Also the standard deviation of the “price_index” is increasing from 0.035 to 0.055. This is confirmed by the two skewed distributions of the “price_index” variable in Figure 1. The share of zeros is large in both years and declines from about 60% zeros in 2013 to about 15% zeros in 2014.^33^

The other variables in Table 1 remain fairly stable between both years. $\;WTP_{j}^{2013}\;$ranges between 1 and 1.5 with a mean of around 1.2 and the $WTP_{ij}^{2014}$ between 1 and 3 with a mean of around 1.3. These differences reflect the different regimes in both years and that markets may have become somewhat more concentrated as less providers were active in the market. In 2013, the variation of market shares of the representative insurer in GHOR regions, $M_{ik}^{2013}$, ranges between 28% and 76% with a mean of 45%. In 2014, the variation of market shares of insurers within providers, $M_{ij}^{2014}$ ranges between 0 and 96% with a mean of 23%. This indicates that some insurers have almost no market share within a provider and that some insurers are very dominant within a provider.

Most providers provide almost all 13 diagnosis categories. On average, almost 8 to 10% of the patients are complex or comorbid. There are however large differences between providers. This holds also for the other variables in Table 1.

Table 2 presents the correlation coefficients. We find a negative correlation between the “price_index” variable and the “WTP” variables in both years suggesting that provider market power plays a role. The insurer market share variables show a small and positive correlation in both years suggesting that insurer market power also plays a role in both years. We find a relatively high negative correlation between WTP measures and complexity and comorbidity measures. This is likely related to the fact that the catchment area of providers with many complex (and comorbid) patients is large, thus these providers have in many micro markets relatively few patients and as a result a lower willingness to pay. Furthermore, we observe some high correlation coefficients among the three control variables. For example, the high correlation coefficient between complexity and comorbidity indicates that patients which are treated by multiple providers also obtain treatments in different diagnoses categories.

**TABLE 2:** Correlation coefficients

	price____index	${\text{WTP}}_{j}^{2013}$	$M_{ik}^{2013}$	CAT_*j*, 2013_	COM_j, 2013_	MOR_*j*, 2013_
**2013**						
price_index	1	‐	‐	‐	‐	‐
${\text{WTP}}_{j}^{2013}$	−0.329^***^	1	‐	‐	‐	‐
$M_{ik}^{2013}$	0.057^***^	−0.110^***^	1	‐	‐	‐
CAT_*j*, 2013_	−0.206^***^	0.504^***^	−0.031^***^	1	‐	‐
COM_*j*, 2013_	0.001	−0.542^***^	0.079^***^	−0.319^***^	1	‐
MOR_*j*, 2013_	−0.035^***^	−0.440^***^	0.067^***^	−0.230^***^	0.753^***^	1
**2014**						
Price index	1	‐	‐	‐	‐	‐
${\text{WTP}}_{ij}^{2014}$	−0.341^***^	1	‐	‐	‐	‐
$M_{ij}^{2014}$	0.110^***^	−0.029^***^	1	‐	‐	‐
CAT_*j*, 2014_	−0.150^***^	0.499^***^	−0.017^***^	1	‐	‐
COM_*j*, 2014_	0.116^***^	−0.442^***^	0.072^***^	−0.314^***^	1	‐
MOR_*j*, 2014_	0.114^***^	−0.405^***^	0.053^***^	−0.317^***^	0.805^***^	1

*Note:* The table shows the Pearson correlation coefficients of the variables presented in Table 1.

^*, **,***^ indicates a significance level (or *p*‐value) of the correlation coefficients at respectively the 0.1, 0.05, and 0.01 level.

## 5 ESTIMATIONS RESULTS

Table 3 presents the results of three different fractional regressions, model (1) refer to 2013 and models (2) and (3) to 2014. In all models, we estimate insurer and treatment fixed effects. In model (3), we included also a cross term between the WTP and market share of the insurer.

**TABLE 3:** Estimation results of three logistic fractional regressions

Dependent variable: price_index			
Year (*t*)	2013	2014	2014
Model	(1)	(2)	(3)
$WTP_{j}^{2013}\;$	−5.807*** (1.091)	‐	‐
$WTP_{ij}^{2014}$	‐	−0.851** (0.368)	−0.747** (0.301)
$M_{ik}^{2013}$	0.688 (0.712)	‐	‐
$M_{ij}^{2014}$	‐	−0.229 (0.144)	0.167 (1.071)
${\text{WTP}}_{ij}^{2014}$ × $M_{ij}^{2014}$	‐	‐	−0.307 (0.845)
*CAT*_*j*,*t*_	−0.0215 (0.0544)	−0.0300 (0.0204)	−0.0299 (0.0205)
*COM*_*j*,*t*_	−7.599** (3.195)	−1.276 (1.555)	−1.325 (1.551)
*MOR*_*j*,*t*_	−4.881 (3.749)	0.837 (2.237)	0.865 (2.211)
Insurer B	−0.494*** (0.160)	−0.447*** (0.119)	−0.448*** (0.118)
Insurer C	−0.211 (0.374)	−0.973*** (0.142)	−0.975*** (0.141)
Insurer D	−0.548*** (0.165)	−1.014*** (0.105)	−1.011*** (0.104)
Insurer E	−0.442*** (0.168)	−1.041*** (0.169)	−1.041*** (0.168)
Insurer F	−0.769*** (0.194)	−1.248*** (0.157)	−1.245*** (0.156)
Insurer FE	Yes	Yes	Yes
Diagnosis FE	Yes	Yes	Yes
Weights	Yes	Yes	Yes
No. of Obs.	21,603	18,400	18,400
Pseudo *R* ^2^	0.0868	0.0492	0.0493

*Note:* All estimations are logistic fractional regressions. Volume per diagnostic codes is used as weights;

A *, **, *** indicate a significance level (or *p*‐value) of the estimated coefficients at respectively the 0.1, 0.05 and 0.01 level. Standard errors are clustered over providers.

**TABLE 4:** Estimation results of two logistic fractional regressions with inverse Loci

Dependent variable: *price_index*		
Year (*t*)	2013	2014
Model	(1)	(2)
$inv\_Loci_{j}^{2013}\;$	−2.659*** (0.532)	‐
$\;inv\_Loci_{ij}^{2014}$	‐	−0.442** (0.190)
$M_{ik}^{2013}$	0.860 (0.727)	‐
$M_{ij}^{2014}$	‐	−0.180 (0.152)
*CAT*_*j*,*t*_	−0.023 (0.055)	−0.025 (0.021)
*COM*_*j*,*t*_	−7.461** (3.196)	−1.506 (1.641)
*MOR*_*j*,*t*_	−4.844 (3.699)	0.976 (2.192)
Insurer B	−0.481*** (0.159)	−0.509*** (0.102)
Insurer C	−0.218 (0.376)	−1.072*** (0.118)
Insurer D	−0.527*** (0.168)	−1.019*** (0.102)
Insurer E	−0.431*** (0.166)	−1.096*** (0.168)
Insurer F	−0.753*** (0.194)	−1.282*** (0.153)
Insurer FE	Yes	Yes
Diagnosis FE	Yes	Yes
Weights	Yes	Yes
No. of obs.	21,603	18,400
Pseudo *R* ^2^	0.088	0.050

*, **, *** indicates a significance level (or *p*‐value) of the estimated coefficients at respectively the 0.1, 0.05, and 0.01 level; standard errors are clustered over providers.

The fractional regression results are interpretable in terms of sign and significance but due to the logistic distribution function the economic impact of coefficients is difficult to interpret. Therefore, we will compute also marginal effects with representative values.

In 2013, we find that the provider market power, in terms of WTP measures, is negative and highly significant. Thus, a provider with a higher WTP has a price that is closer to the maximum price. This significant result is quite remarkable as most prices are equal or close to the maximum price. The coefficient of the market share of the largest insurer in the region ($M_{ik}^{2013}$) is positive, that is a larger market share results in a larger price deviation from the maximum price. The effect is, however, not significant. The control variables are insignificant but have the expected negative sign. For example, if a provider has more comorbid patients it charges a price that is closer to the maximum price. As is clear from the insurer dummies, the different insurers pay different prices.

In both 2014 models, the provider market power (WTP) is negative and significant at the 5% level. We find positive and negative values for the market share of the individual insurers $M_{ij}^{2014}$. The coefficients are however not significant at the 5% level. Also the interaction term is not significant. The control variables are not significant. Also in 2014, the insurer dummies indicate that insurers vary in the way they set their prices.

**FIGURE 2:**
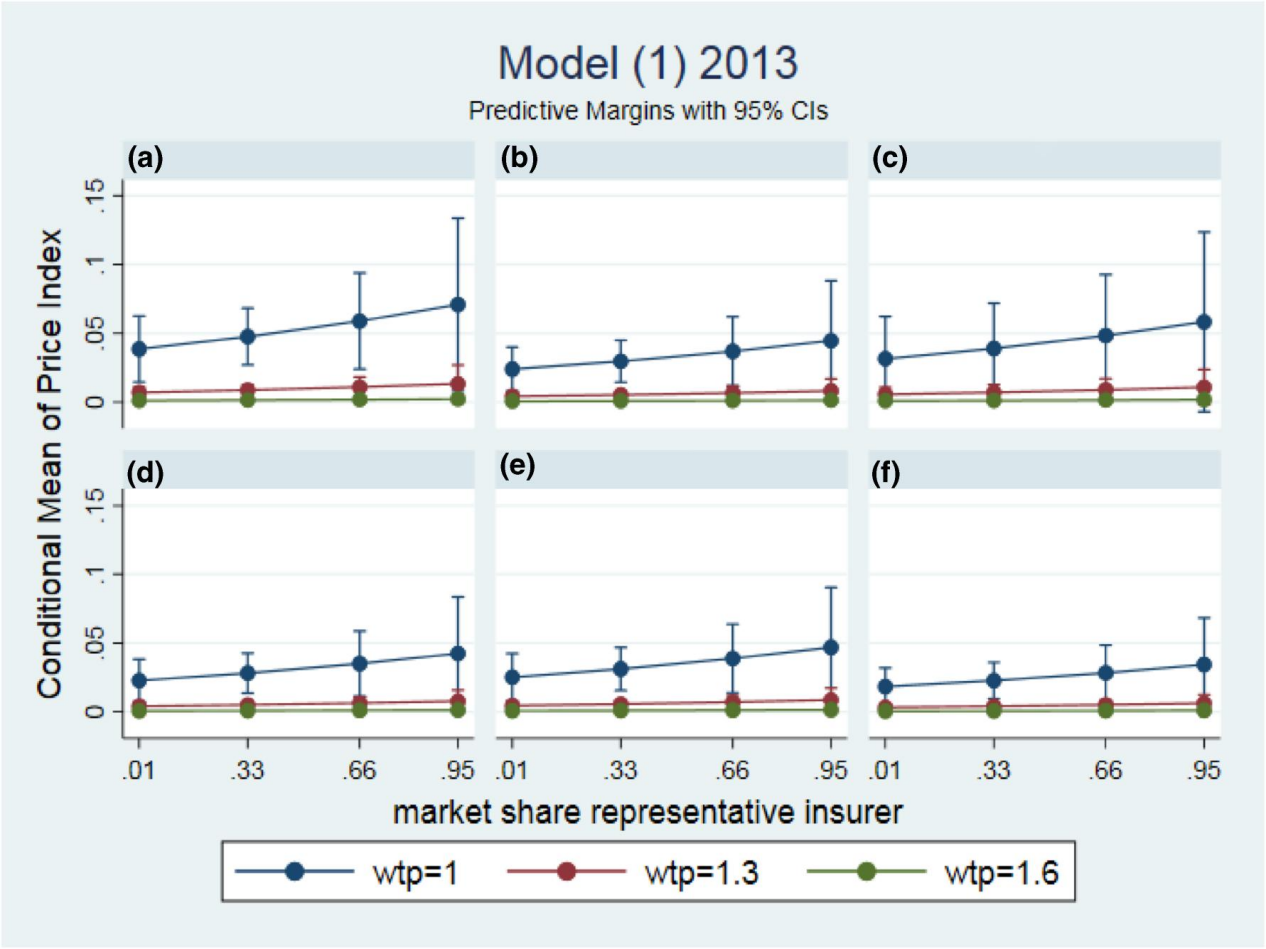
Marginal effects of representative values of provider market power ($\mathrm{WT}P_{j}^{2013}$) and different market shares of representative insurers ($M_{\mathrm{ik}}^{2013}$)

To interpret the economic size of the effects, we plotted in Figure 2 the estimated marginal effects for 2013 of model (1) in six different panels that each represent a different representative insurer for a range of WTP values (${\text{WTP}}_{j}^{2013}$) and insurers' market shares ($M_{ik}^{2013}$). The figure shows for all six insurers average price effects, that are between 0 and about 6% lower than the regulated maximum price in 2013. Insurers A and C have negotiated somewhat lower contract prices (and a higher price_index) than insurers B and D till F, with insurer F having the highest contract prices. A higher WTP‐value corresponds with a contract price closer to the regulated maximum price, which suggests that providers with more market power negotiate higher contract prices. For example, for a provider with a high WTP value of 1.6, the average price effect is 0.1%, which indicates that almost all prices of this provider equal the maximum price. The market share of a regional insurer does hardly influence this price‐effect. This differs for providers with a lower WTP.

For example, for a low WTP‐value of 1 and a representative insurer with a market share of 25%, we have price effects that range between about 3% and 4%, and for an insurer with a market share of 75% we find a range between about 4% and 5% lower than the maximum price. However, these insurer effects are all insignificant. To conclude, in 2013, we find that providers with more market power are able to negotiate higher prices but the effects are relatively small.

**FIGURE 3:**
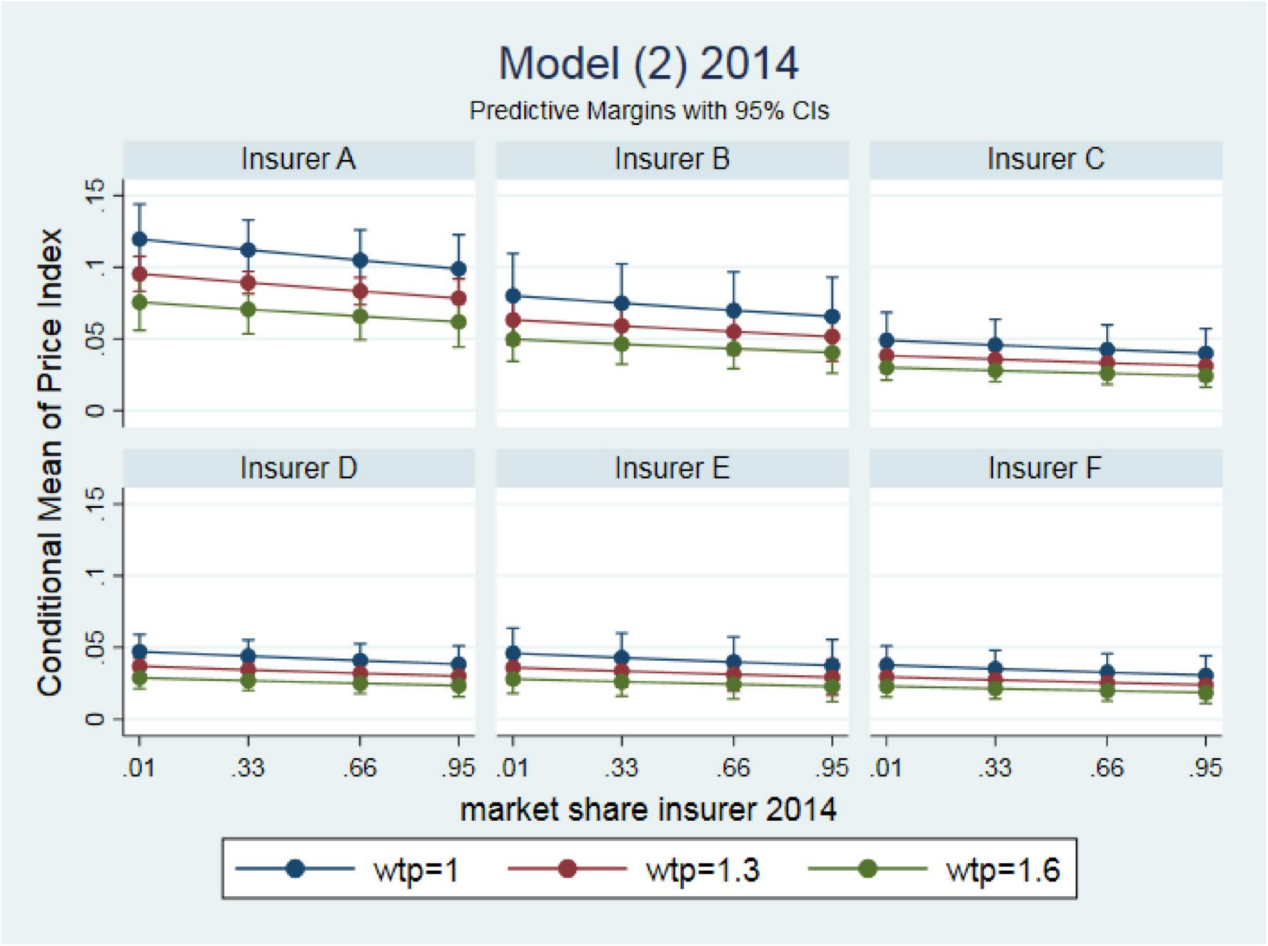
Marginal effects of representative values of provider market power ($\mathrm{WT}P_{\mathrm{ij}}^{2014}$) and different market shares of insurers ($M_{\mathrm{ij}}^{2014}$)

Figure 3 shows the estimated effects for 2014 of model (2) in six different panels that each represent a different insurer. The price‐effects are now much larger than in 2013 and ranges for providers with a small WTP‐value of 1 from about 3.1% to 12.0% lower than the regulated maximum price. For providers with a relatively large WTP‐value of 1.6 price.

Effects range from about 1.9% to 7.6% lower than the regulated maximum price. In contrast to 2013, a larger market share of an insurer in 2014 leads to a price closer to the regulated maximum price, although the effects are small and insignificant. As is clear from Figure 3, there is large variation across insurers. The pattern is quite similar to 2013, insurers A and B have negotiated much lower contract prices (and therefore a higher price index) than insurers C till F, with again insurer F having the highest contract prices. For instance, the estimated price index for insurer A range from about 6.2% to 12.0% and for insurer F from 1.9% to 3.8% lower than the maximum price. These findings indicate that some insurers took the opportunity to negotiate lower prices than the regulated maximum prices. However, these effects are unrelated to an insurers' market share. This is confirmed by the insignificant Spearman rank correlation coefficient of 0.09 between the estimated effects of the insurer dummies and the insurers' national market shares. Our finding could indicate that different insurers follow different strategies. First, it could be that some (smaller) insurers simply put more effort in negotiating lower contract prices with providers than other (larger) insurers. Second, the “paradox of power” could play a role (Hirshleifer, 1991). It may be easier for small insurers to set up preferred provider networks and firmly negotiate on prices, while large insurers have to fulfill their duty of care and therefore are obliged to contract all providers in a market.^34^ Third, it could be that some insurers put less effort in price negotiations and more effort in budget negotiations.^35^

In Appendix A, we show the figures for models (3). As a robustness check, we also run a regression with the inverse Loci as a market power indicator (Capps et al, 2003; Gaynor, Kleiner, & Vogt, 2013). The results are very similar to the ones with the WTP as market power indicator (see appendix B).

## 6 CONCLUSION

We find that Dutch mental health providers with more market power, that is, a higher willingness to pay measure, contracted significantly higher prices. In general, such effects may be small or not visible when maximum prices are set by a regulator. For example, if the regulator sets maximum prices at or just below cost‐prices then contract prices will have to be close to the maximum price as otherwise providers cannot sustain in the market. However, if we observe that all prices are close to the maximum price then this does not necessarily mean that maximum prices are close to cost‐prices, it could also indicate that contract prices are well above cost‐price because providers have strong market power vis‐à‐vis insurers. In the case of maximum prices, we can only measure the balance of power if sufficient contract prices are set below the maximum price.

We study a major policy change in 2014 in Dutch mental health care where the regulator increased maximum prices by about 10%. Moreover, in the same year, there was a profound change to managed competition and all mental health care providers had to negotiate for the first time individually with all health insurers in the market about prices for mental health services. Since most of the negotiated contract prices were set below the maximum price, we use this policy change as a quasi‐experimental design to study the balance of powers between insurers and providers.

Although, in 2013, most contract prices equaled the maximum price, we still find that providers with more market power were able to negotiate prices on or closer to the maximum price with regional insurers. Since most contract prices are close to the maximum price, price differences across providers with high and low market power are small. In 2014, when there became more room to negotiate about prices, we find that mental health providers with more market power, that is, a higher WTP measure, contracted significantly higher prices. Depending on the type of insurer, price‐effect ranges for providers with weak market power from 3% to 12% lower than the regulated maximum price and for providers with strong market power from 1% to 6% lower than the regulated maximum price. The fact that the majority of the contract prices are set below the maximum price provide evidence that insurer market power plays a role and suggests that competition works in setting lower prices, especially if providers have low market power. If a provider has a higher WTP, the contract prices are significantly higher and the mitigating effect of the insurer's market share is less strong. A lower price per treatment allows for more treatments within the budget. For example, in a regime as in 2013, prices per treatment would probably be higher, that is, closer to the maximum price, with greater chances to overspend the budget.

Some insurers negotiated significantly lower prices than other insurers but these differences are not related to an insurers' market share. Price negotiations are complex when there exists a mutual but asymmetrical dependency relation between providers and insurers in a region. It is likely that in the first year of individual competition, insurers still had to adapt to the new situation. For example, some insurers adapt more quickly and put more effort in negotiating contract prices with providers than other (larger) insurers. It may also be related to the fact that insurers are all not‐for profit with different objective functions. For example, some (larger) insurers may be less profit oriented than others. Another argument that could play role is the “paradox of power”, large insurers have to fulfill their duty of care and therefore are obliged to contract all providers in a market. As a result, it may be harder to firmly negotiate on price when an insurer is larger. It also it could be that some insurers put less effort in price negotiations and more effort in budget negotiations. More research is needed to find out which of these explanations play an important role.

Our study has limitations. Although the policy change is an ideal experiment to measure price setting behavior of provider‐insurer pairs for the first time in a new “managed competition” environment it is not clear to what extent our results are generalizable to later years. The strategies that some providers or insurers use in 2014 are first year strategies which may be adapted over time.^36^ Illustrative might here be a lawsuit in 2016 where insurers argued convincingly that the maximum prices set by the regulator in 2014 (and also in 2015) was set too high and not based on decent cost‐price calculations.^37^ The regulator lost the lawsuit and had to recalculate cost‐prices and lower maximum prices for 2014.

Another limitation of our statistical analyses is that the market consists of relatively few health insurers. With few health insurers, an outlier strategy of one insurer can already have a substantial impact on the results.

DTC‐prices may not always be correctly specified in the database. For example, consider a situation where providers hit the budget ceiling earlier in the year and perform some “overproduction” of treatments for which they do not receive additionally payment. In the database we do not observe for these additional treatments a zero price, but the same price for similar DTC's. It is not clear to the researcher whether this price is the ex‐ante negotiated price, or the correctly specified ex‐post updated price. However, we expect the impact for our analyses to be small.^38^

Contracts may differ at other unobservable dimensions. For example, quality or performance incentives may have played a role in provider‐insurer negotiations (Ruwaard, 2018). However, it is unlikely that quality or performance incentives have played a large role, as 2014 was the first year of individual competition and insurers and providers still had to learn how to design these type of contracts.

## CONFLICT OF INTEREST

The authors have declared that they have nothing to disclose.

## Figures and Tables

**FIGURE 4:**
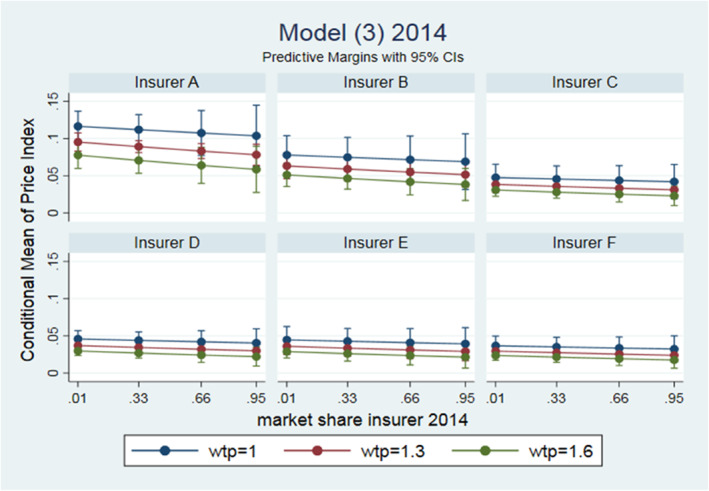
Marginal effects of representative values of provider market power (${\text{WTP}}_{j}^{2013}$) and different market shares of representative insurers ($M_{\mathrm{ik}}^{2013}$) for model (3) in Table 3

**FIGURE 5:**
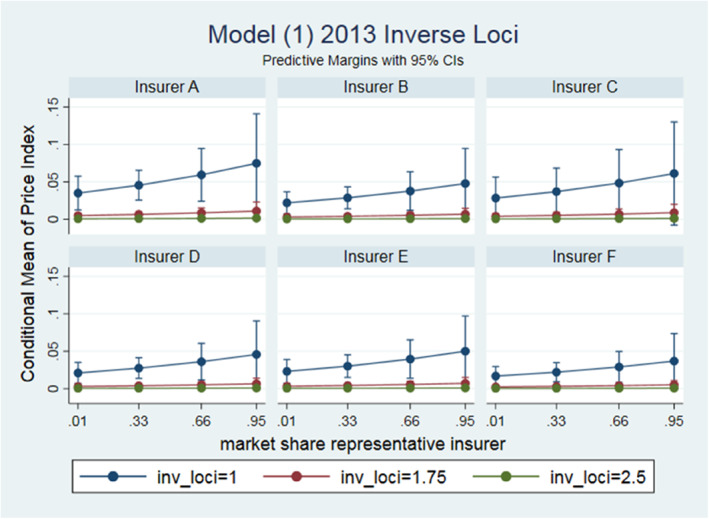
Marginal effects of representative values of provider market power (inv_$\mathrm{Loc}i_{j}^{2013}$) and different market shares of representative insurers ($M_{\mathrm{ik}}^{2013}$) for model (1) in Table 4

**FIGURE 6:**
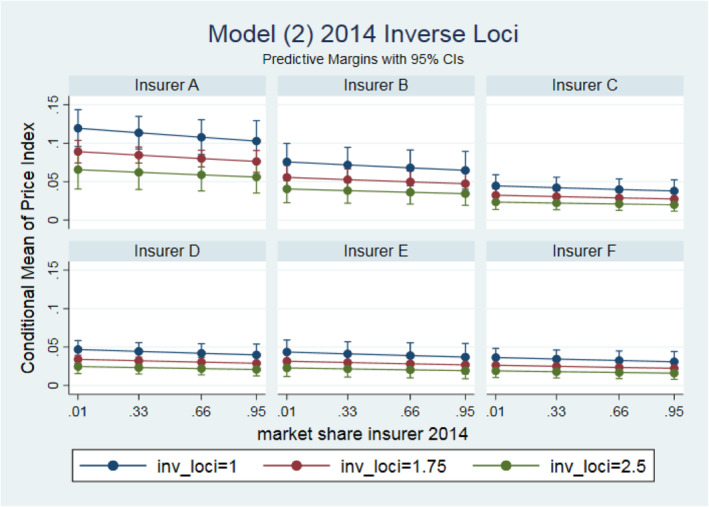
Marginal effects of representative values of provider market power ($\mathrm{inv}\_\mathrm{Loc}i_{j}^{2014}$) and different market shares of insurers ($M_{\mathrm{ij}}^{2014}$) for model (2) in Table 4

## ENDNOTES

^2^ Merger studies suffer from selection effects as the merger itself is not an exogenous event.

^3^ This identification strategy is similar to Clemens and Gottlieb (2014) who use as exogenous event sudden adjustments of provider reimbursement rates in US Medicare.

^4^ To explain contract prices we take also cost‐price measures into account, but it is unclear how important cost prices are in the price negotiation process. In the case of maximum prices, especially providers may use the maximum price as a reference point in their negotiations. We have no information about the use of quality indicators in contracts, but quality indicators were rare in the negotiations in 2014 (Ruwaard, 2018).

^5^ In the Netherlands, there are four large insurers who have together a market share of about 90%. The insignificant results suggest that at least one or two of these large insurers were less price oriented.

^6^ Many health insurance companies have different brands but purchasing care occurs on the company level.

^7^ For a more extensive description of the insurer market before and after the reform, see Douven, Katona, Schut, and Shestalova (2017).

^8^ For privacy reasons we anonymize these six insurers in our data.

^9^ The DTC is similar to a Diagnostic‐Related Group (DRG) that is used in many other countries. See Westerdijk, Zuurbier, Ludwig, and Prins (2012) for an extensive discussion on DTCs.

^10^ The regulator is the Dutch Health Care Authority (NZa).

^11^ Only in very special circumstances, for very complex patient groups, the DTC price was allowed to be higher than the maximum price. Higher prices were capped by so called max‐max prices. Max‐max prices can be set at most 10% above the maximum prices. As these max‐max prices are rare, and the extra costs have to be substantiated, we did not include these max‐max prices in our research.

^12^ The remaining 10% was carried out by self‐employed psychologists and psychiatrists. We excluded these providers in our analysis because they are much smaller in size and were placed already in 2008 under a different regime.

^13^ These budgets were based on expected case mix, several regional budget parameters (such as inflation, wages, capital costs etc.) and the production in the previous year. The determination of this budget is a complex process which may also be based on possible future transitions and growth expectations.

^14^ In 2014, about 71% of all treatments were contracted with ex‐ante prices and a budget that serves as a ceiling. In the other 29% of the contracts prizes were mainly calculated ex‐post such that the sum of the prices of all treatments produced in a year equals the budget (NZa, 2014). Also in the latter case the ex‐post calculated price is a good reflection of the value of a treatment.

^15^ Mental health providers that hit the budget ceiling may refer patients to other providers. Some providers and insurers determined their DTC prices retrospectively, after annual production is known (NZa, 2016).

^16^ There were 26 different geographical regions (GHOR‐regions) that were originally designed for providing medical assistance in case of accidents, disasters and crises (for more information about these regions, see www.ghor.nl [in Dutch]).

^17^ Thus, selective contracting was not allowed.

^18^ Insurers incurred also somewhat more risk in 2014, compared to 2013, because of a reduction in the ex‐post payment scheme in the risk adjustment system (NZa, 2015). Also in 2014, all insurers contracted all providers.

^19^ In 2012 and 2013 the regulator performed a large investigation to the cost‐price calculation of DTC prices, which resulted in an overall increase of DTC‐prices.

^20^ On the other hand, the representative health insurer was essentially a regional monopsonist and may have had more buying power than the individual insurers that operated in the more competitive market in 2014.

^21^ For example, insurers and providers negotiate about a DTC for “depression” within a duration range “250 till 799 min”. All the DTCs that fall within this category have the same contract price, regardless the individual patients' characteristics. Using a group of DTCs as unit of observation implies that we weight all groups equally, that is independent of the number of DTCs within a group.

^22^ Most mental health care providers operate regionally. In those cases that a provider operates in more than one region we use for each region the same WTP measure. Differentiating WTP measures across (mostly adjacent) regions could result in unintended large variations in WTP measures for the same provider which does not seem plausible. Moreover, insurers operate nationally and may also be dominant in adjacent regions (and negotiate with the same provider in those regions as well).

^23^ Note that Gowrisankaran et al (2015) also allow for insurance specific provider market power measures.

^24^ We run for 2013 also our regression with as market power indicator the individual market share of each insurer in the GHOR‐region as well as including only the observations of the representative insurer. In the latter case, the number of observations decline (*n* = 5328). The results were almost the same. These results are available upon request.

^25^ There are thirteen different diagnoses, such as “depression”, “anxiety”, “schizophrenia”, and so on. A provider with fewer diagnosis categories may be more specialized. A specialized provider may be more efficient, and thus cheaper, but may also treat more complex patients, and thus be more expensive.

^26^ Differentiating control variables across insurers and regions can result in large unintended cost variations within the same provider which does not seem plausible.

^27^ A Logistic regression is less suitable as the endpoints 0 and 1 should be included. A Tobit approach works best when there are many observations at both ends of the unit interval. This is not the case in our data, which is left censored at zero. Furthermore, the endpoints are the results of bargaining processes between an insurers and providers and not the result of some type of censoring making a Tobit model less suitable. For a discussion, see Ramalho et al. (2011).

^28^ We perform our regressions with the “fracreg” command in STATA.

^29^ For example, some insurers may simply put more effort in their price negotiation process with providers than others, irrespective of their market power.

^30^ The data consists of about 400,000 DTCs in 2013 and 340,000 in 2014.

^31^ The reason is that contract prices were in most cases the same for each mental health service with the same diagnosis belonging to a specific insurer‐provider combination.

^32^ Each provider is coded with a unique id number in our data. We do not observe provider networks.

^33^ Figure 1 shows that markets with maximum prices seems to results in much lower price variation than markets without maximum prices. For example, recently Cooper et al. (2019) and Douven, Burger, and Schut (2020) study hospital markets where insurers and hospitals can freely negotiate about contract prices and they report much larger variations in contract prices.

^34^ See also Ho and Lee (2017).

^35^ An insurer with market power might not negotiate on prices but opt for a low volume of treatments combined with a fixed budget. If an insurer succeeds, prices might be higher than average which we would incorrectly measure as low market power in our analyses. We don't know whether this strategy has occurred in our analyses. It is a risky strategy as it may lead to longer waiting lists and renegotiations about the budget at the end of the year for additional production.

^36^ When more data becomes available we can test these issues.

^37^ For more information, see https://uitspraken.rechtspraak.nl/inziendocument?id=ECLI:NL:CBB:2017:402 (in Dutch).

^38^ We expect “overproduction” to be limited. When providers hit the budget ceiling they can refer patients to other providers that still have room in the budget to treat patients, or put patients on a waiting list (Algemene Rekenkamer, 2017). Also, when it becomes clear that the budget hits the ceiling providers have an incentive to renegotiate about additional budget, keeping the difference between average ex‐ante contract prices and ex‐post updated contract prices small.

## APPENDIX A

### A.1

In this appendix, we show the marginal effects that correspond to the estimation results of equation (3) in Table 3. Figure 4 is very similar to Figure 3 in the main text.

## APPENDIX B

### B.1

In this appendix, we show the results of the same estimations as in the text but for an alternative market power indicator, the inverse Loci. The Logit competition index (LOCI) is an indicator for a provider's market power. The indicator results from profit maximization with respect to price subject to logit demand under differentiated Bertrand competition. Theoretically, the higher the inverse Loci, the higher the price‐cost margin of the provider. For more information and how to compute the inverse Loci we refer to Akosa Antwi, Gaynor, and Vogt (2006), Gaynor and Town (2012), NZa (2013b) and Berden et al. (2019). Table 4 and Figures 5 and 6 present similar results as for models (1) and (2) in Table 3 where we replace in 2013 the inv_${\text{Loci}}_{j}^{2013}$ for ${\text{WTP}}_{j}^{2013}$ and in 2014 the ${\text{inv\_Loci}}_{ij}^{2014}$ for ${\text{WTP}}_{ij}^{2014}$. The results of the inverse Loci‐models are very similar to the results in Table 3.
